# Axicabtagene ciloleucel CD19 CAR-T cell therapy results in high rates of systemic and neurologic remissions in ten patients with refractory large B cell lymphoma including two with HIV and viral hepatitis

**DOI:** 10.1186/s13045-019-0838-y

**Published:** 2020-01-03

**Authors:** Ahmed Abbasi, Stephen Peeke, Nishi Shah, Jennat Mustafa, Fariha Khatun, Amanda Lombardo, Michelly Abreu, Richard Elkind, Karen Fehn, Alyssa de Castro, Yanhua Wang, Olga Derman, Randin Nelson, Joan Uehlinger, Kira Gritsman, R. Alejandro Sica, Noah Kornblum, Ioannis Mantzaris, Aditi Shastri, Murali Janakiram, Mendel Goldfinger, Amit Verma, Ira Braunschweig, Lizamarie Bachier-Rodriguez

**Affiliations:** 1 Department of Oncology, Montefiore Medical Center, Albert Einstein College of Medicine, The Bronx, NY USA; 2 Department of Pharmacy, Montefiore Medical Center, The Bronx, NY USA; 3 Department of Pathology, Montefiore Medical Center, The Bronx, NY USA

**Keywords:** CD19 CAR-T, HIV and CD-19 CAR-T, CNS and CD-19 CAR-T, Axi-cel, Hepatitis B and CD-19 CAR-T

## Abstract

Axicabtagene ciloleucel (Axi-cel) is a CD-19 Chimeric Antigen Receptor T cell therapy approved for the treatment of relapsed/refractory diffuse large B cell lymphoma. We treated ten patients with DLBCL post-FDA approval in an inner-city tertiary center in the Bronx. Eight patients (80%) had received ≥ 3 lines of therapy, six patients had received prior radiation, and seven had recurrent disease after prior autologous hematopoietic stem cell transplant (AHCT). Our cohort included one patient with HIV, two patients with hepatitis B, and two patients with CNS involvement of lymphoma. Axi-cel treatment led to significant responses with 8/10 patients achieving a complete remission at 3 months, including both patients with prior CNS involvement. The treatment was generally well tolerated with 20% of patients experiencing grade ≥ 2 CRS. One patient each with HIV and hepatitis B responded without significant toxicities. In conclusion, Axi-cel led to significant efficacy with manageable toxicity in DLBCL in a real-world setting.

To the Editor,

CD-19 Chimeric Antigen Receptor–T (CAR-T) cell therapy is FDA approved for the treatment of refractory or recurrent (R/R) diffuse large B cell lymphoma (DLBCL). The two commercially available cellular products, axicabtagene ciloleucel [Axi-cel] and Tisagenlecleucel have shown complete response in 54% of 111 and 40% of 93 patients, respectively, in clinical trials [1–4]. The most concerning side effects are cytokine release syndrome (CRS) and immune effector cell-associated neurotoxicity syndrome (ICANS) [4–6]. We evaluated efficacy and safety of Axi-cel post-FDA approval in an inner-city tertiary medical center.

We retrospectively reviewed the first ten R/R DLBCL patients that were treated with Axi-cel between June 2018 and June 2019 at our center. We collected laboratory and clinical parameters and responses post-CAR-T by PET/CT. All patients received lymphodepleting chemotherapy per product guidelines with fludarabine and cyclophosphamide [2].

Patient disease characteristics including high-risk features (rearrangements or expression of BCL-2, MYC, BCL-6) and treatment characteristics are summarized in Table 1. Four patients had non-GCB DLBCL and four had transformed from prior low-grade disease (three follicular lymphomas and one lymphoplasmacytic lymphoma). Six patients had received prior radiation, and seven had prior autologous stem cell transplant. Two patients had prior CNS involvement. One had received intrathecal (IT) methotrexate 3 months prior to CAR-T. The other patient had a history of leptomeningeal disease and sixth cranial nerve palsy previously treated with high-dose methotrexate and IT chemotherapy. The latter patient had disease recurrence in the left nasopharynx prior to CAR-T infusion. Two patients had active HBV infection and were on anti-viral treatment. One patient had HIV and was on anti-retroviral therapy with undetectable viral load prior to therapy with CD4 count of 127 cells/μl.

**Table 1:** Patient and treatment characteristics

Patient characteristics	*N* = 10
	*n* (%)
Median age (range) years	66 (55–77)
Female gender	5 (50%)
ECOG performance status ≥ 2	2 (20%)
Ethnicity	
Hispanics	4 (40%)
White	3 (30%)
African American	2 (20%)
Asian	1 (10%)
Disease characteristics	*n* (%)
Non-GCB DLBCL	4 (40%)
Transformed lymphoma	3 (30%)
Double expressor lymphoma	6 (60%)
Triple expressor lymphoma	2 (20%)
Double hit lymphoma	1 (10%)
Triple hit lymphoma	1 (10%)
P53 deletion by FISH	1 (10%)
Disease stage	
Stage I	0
Stage II	1 (10%)
Stage III	4 (40%)
Stage IV	5 (50%)
Number of prior lines of therapy	
1	0
2	2 (20%)
3	3 (30%)
4	5 (50%)
CNS involvement	2 (20%)
Prior AHCT	7 (70%)
Response to treatment	*n* (%)
CR at 3 months	8 (80%)
POD	1 (10%)
Adverse events	
CRS	6 (60%)
CRS grade ≥ 2	2 (20%)
ICANS	5 (50%)
ICANS grade ≥ 2	3 (30%)
Tocilizumab	3 (30%)
Glucocorticoids	1 (10%)
Neutropenia (< 500 c/μl)	10 (100%)
Neutropenic fever	8 (80%)
Thrombocytopenia (< 50,000 c/μl)	7 (70%)
Documented infection	5 (50%)

Post-CAR-T, patients had a peak in temperature at ~ day 5. All patients developed neutropenia (absolute neutrophil count (ANC) of < 500 c/μl) with duration of ~ 6 days and ANC nadir occurred on day 6 post-infusion. Neutropenic fever occurred in 8/10 patients. Platelet count nadir and maximum CRP elevation also occurred at around day 6. Lymphocyte depletion was achieved in all patients prior to CAR-T. Lymphopenia lasted 2 weeks, with lymphocyte recovery seen at day 12. Mean length of hospital stay was 22.8 days.

CRS and ICANS were evaluated and graded per ASTCT guidelines [7]. CRS was seen in six patients (≥ grade 2 CRS in two). ICANS was observed in five patients (≥ grade 3 ICANS in three). Three patients received tocilizumab. Two patients developed *Clostridium difficile* colitis, one of whom required a MICU stay for hypotension with requirement of one pressor for < 48 h (grade 3 CRS) and eventually had full recovery.

Figure 1 shows responses and follow-up for all treated patients (data cutoff October 15, 2019). Eight of 10 patients achieved a CR including the two patients with prior CNS involvement, HIV patient, and one patient with HBV, as assessed by PET/CT (Fig. 2). Additional file 1: Table S1 shows viral load information on HIV and HBV patients.

**Fig. 1:**
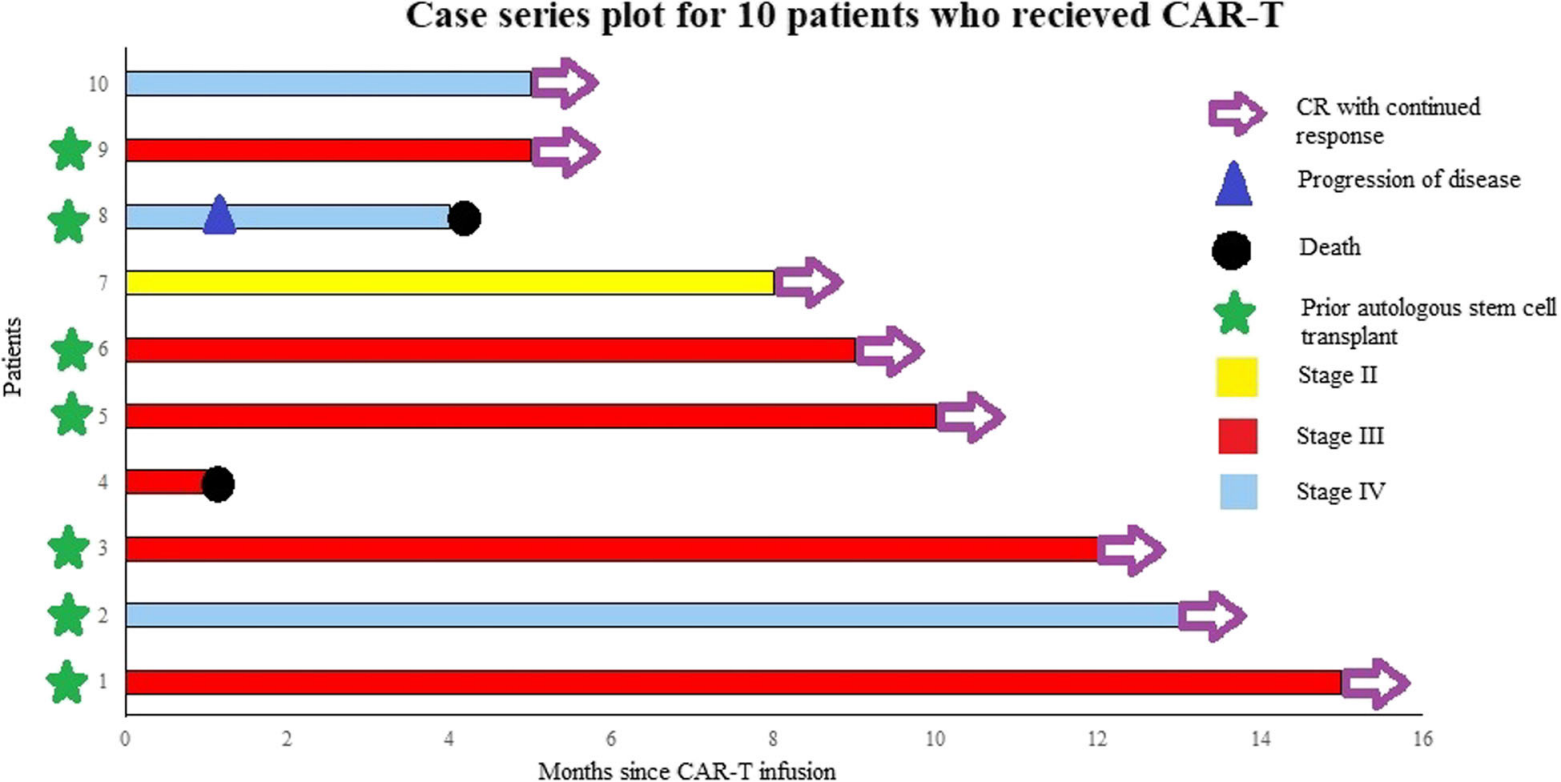
Response to treatment after axicabtagene ciloleucel. Swimmer’s plot showing response to treatment after Axi-cel infusion in patients with DLBCL receiving CD19-directed CAR-T

**Fig. 2:**
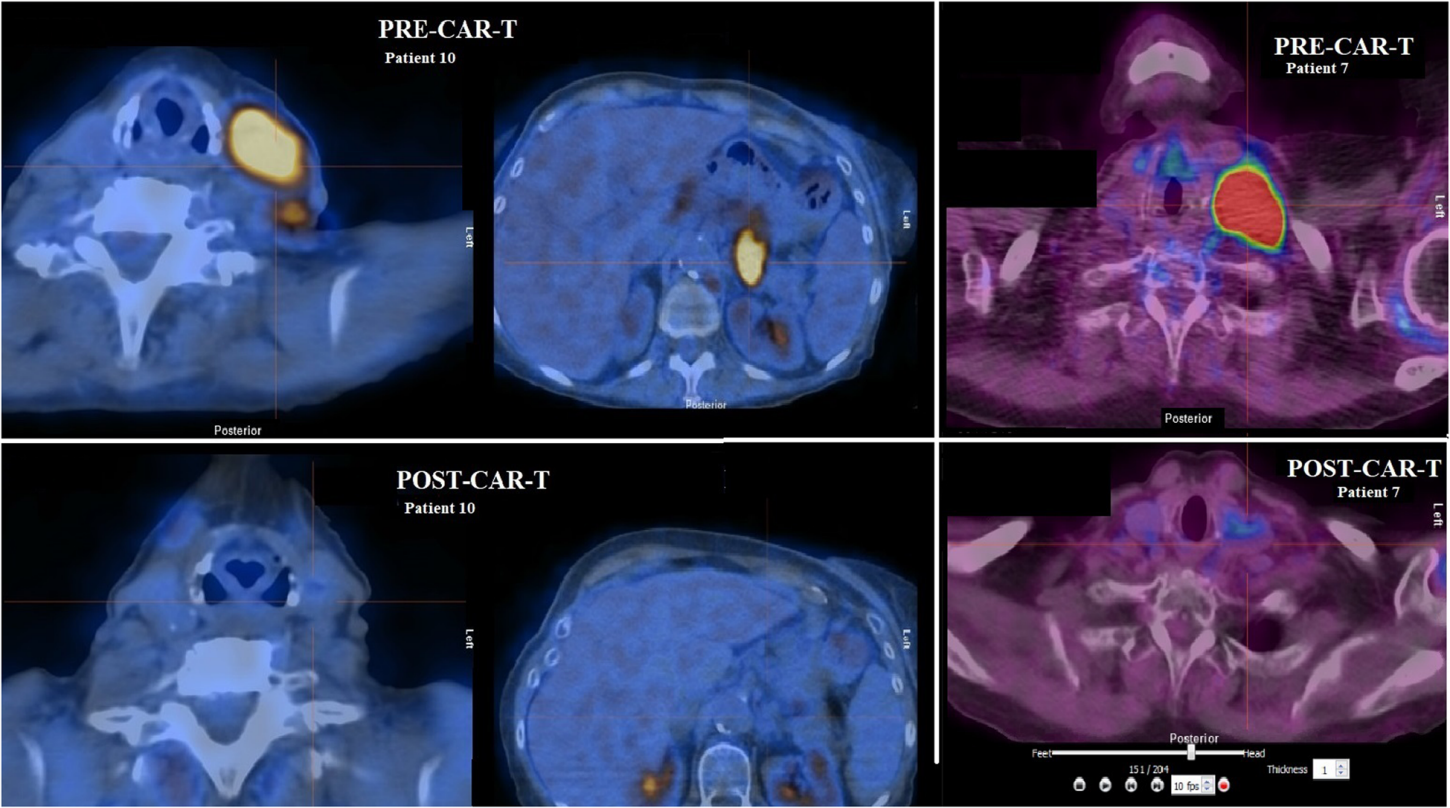
PET-CT images of patients treated with axicabtagene ciloleucel. Pre-CAR-T (above) ad post-CAR-T (below) images show improvement in left cervical and left paraaortic lymphadenopathy in patient 10 and improvement in left cervical lymphadenopathy in patient 7

One patient had clear progression of disease (PD) within a month of treatment and died 4 months later due to complications of his disease. His lymphoma had transformed from prior low-grade disease and had acquired a TP53 deletion. He was positive for hepatitis B core Ab.

One patient developed severe neurotoxicity, requiring tocilizumab and glucocorticoids, and expired during hospital stay. This patient had an aggressive, triple expressor lymphoma (BCL-2, BCL-6, and MYC) with widespread extra-nodal disease but no prior CNS involvement. Brain imaging prior to death showed vasogenic edema and mass effect suspicious for mass, but given the patient’s altered mental status, a high-quality diagnostic study was not obtained. CSF cytology was negative for malignant cells. Another patient who developed grade 1 ICANS post-CAR-T had a CR on PET/CT but remains with significant cognition issues post-treatment at last follow-up.

Our data supports the feasibility of delivering CAR-T to heavily pre-treated patients within a reasonable time frame: ~ 1 month from collection to infusion and 2–3 weeks of hospitalization during treatment. Our series show severe episodes occurred in < 25% of patients which is lower than previously reported.

It is encouraging to see the high rate of CR in heavily pretreated patients in a real-world setting especially for those with CNS involvement, HIV, and active HBV as these patients were excluded from the original clinical trial. The two deaths seen in our cohort were related to lymphoma progression (one confirmed on PET/CT and another patient who had CNS imaging concerning for mass effect vs. vasogenic edema post-CAR-T). The potential for longer-term adverse effects, as seen in the cognitive issues post-treatment for one patient, warrants continued monitoring.

In conclusion, our study adds to the mounting evidence of the safety and efficacy of CD-19-directed CAR-T therapy in aggressive DLBCL patients with severe comorbidities (CNS involvement, HIV, HBV) in a real-world setting [8–10].

## Supplementary information

**Additional file 1: Table S1.**. Viral load and other lab parameters for three patients (1 HIV, 2 patients with HBV).

## Data Availability

All data generated or analyzed during this study are included in this published article.

## Abbreviations

AHCT: Autologous hematopoietic stem cell transplant
ALL: Acute lymphoblastic leukemia
ANC: Absolute neutrophil count
ART: Anti-retroviral therapy
CAR-T: CD-19 Chimeric Antigen Receptor–T
CR: Complete response
CRS: Cytokine release syndrome
DEL: Double expressor lymphoma
DLBCL: Diffuse large B cell lymphoma
ICANS: Immune effector cell-associated neurotoxicity syndrome
IHC: Immunohistochemistry
IT: Intrathecal
TEL: Triple expressor lymphoma
